# Snapshot on a Pilot Metagenomic Study for the Appraisal of Gut Microbial Diversity in Mice, Cat, and Man

**DOI:** 10.1155/2016/6587825

**Published:** 2016-04-24

**Authors:** Coline Plé, Louise-Eva Vandenborght, Nathalie Adele-Dit-Renseville, Jérôme Breton, Catherine Daniel, Stéphanie Ferreira, Foligné Benoît

**Affiliations:** ^1^Université Lille, CNRS, Inserm, CHU Lille, Institut Pasteur de Lille, U1019, UMR 8204, Centre d'Infection et d'Immunité de Lille, 59000 Lille, France; ^2^Genoscreen, Service of Research, Development and Innovation in Health and Environment, 1 rue du Pr Calmette, BP 245, 59019 Lille, France

## Abstract

Gut microbiota plays a key role in the maintenance of homeostasis and host physiology, comprising development, metabolism, and immunity. Profiling the composition and the gastrointestinal microbiome with a reliable methodology is of substantial interest to yield new insights into the pathogenesis of many diseases as well as defining new prophylactic and therapeutic interventions. Here, we briefly present our methodology applied to fecal samples from mice and then further extended to the samples from a cat and a single human subject at 4 different time points as examples to illustrate the methodological strengths. Both interindividual and time-related variations are demonstrated and discussed.

## 1. Introduction

Recent developments in metagenomics have provided researchers with the tools needed to open the “black box” of microbiome science. These novel technologies have enabled the establishment of correlations between dysbiotic microbial communities and many diseases. Extended approaches and meticulous data interpretation will be important for resolution of these discrepancies. In this context, diagnostic tools and analytic solutions for research purposes are needed to support clinical studies in humans and preclinical developments using mice. The growing need to survey the tremendous microbial diversity in a culture independent manner has led to the development of molecular methods through sequence profiling of part of conserved genes such as 16S rDNA, in various scientific fields including ecology (plants, animals), agronomy, biotechnology, and of course human health. Next-generation sequencing technologies providing unprecedented throughput of data are now routinely used to assess bacterial community composition in complex samples. Depending on whether rough/basic bacterial signature or extensive resolution of taxonomic assignment of organisms is needed, the time and costs for 16S rRNA profiling* versus* full genome analysis or bacterial RNA sequencing may vary from 1 to 50.

## 2. Materials and Methods

**Figure 1:**
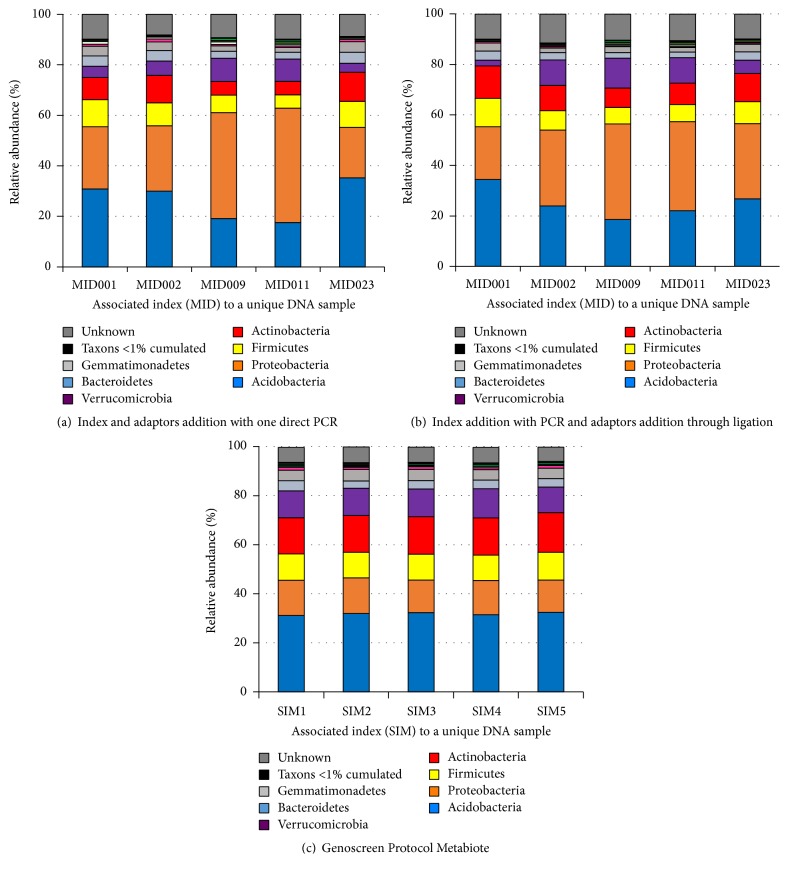
Impact of three different 16S-amplicon library preparation methods on the observed relative abundance of bacterial taxa. One unique sample of soil (known to have the highest microbial community complexity) was used to compare three distinct methods of molecular indexing such as MID (multiplex identifier) or Genoscreen-developed sample identifier multiplex (SIM). Five examples of final bacterial profiling are shown for each of the three methods used to illustrate the reproducibility of DNA extraction.

**Figure 2:**
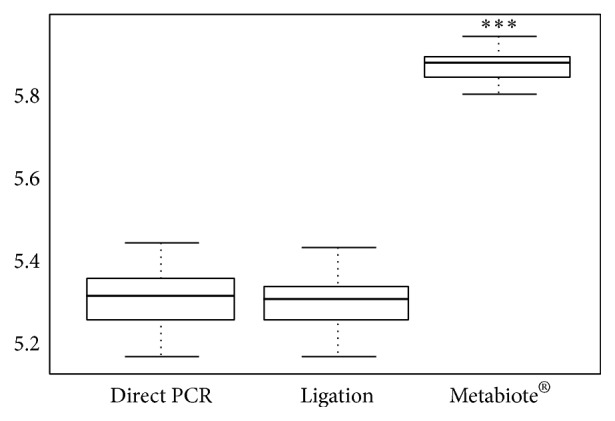
Shannon diversity index obtained with the three different protocols of 16S-amplicon library preparation. *N* = 48 distinct indexes were used to tag a unique sample (from soil) for each of the three methods of molecular indexing. The Metabiote indexing system reveals an extended diversity reached, ^*∗∗∗*^ *P* < 0.001, and Mann-Whitney test.

The Roche 454 GS FLX and GS Junior Sequencing Systems have been employed by researchers worldwide to accurately characterize diverse microbial communities, as demonstrated in the over 1,000 metagenomics publications to date. However, several protocols for amplicon-based sequencing of 16S rRNA still exist and are widely used to perform these analyses whereas no study has looked at their respective impact on taxonomical description, relative abundance of taxa, and diversity and richness indexes. A comparison of two classical amplicon library preparations (Direct PCR and Ligation) has been performed by Genoscreen (Lille, France) which led them to develop an optimized and standardized solution for the analysis of microbiota named Metabiote. Indeed, starting with DNA extracted from one unique sample of soil (known to have the highest microbial community complexity), several identical libraries (*n* = 48) were prepared with distinct molecular tags as indexes namely MID (standing for multiplex identifier), following two classical protocols (resp. amplification and ligation) and the developed Metabiote® Protocol, using an own molecular index system, namely, sample identifier multiplex (SIM). As an example using 5 different MID (from the 48 items), Figure 1 clearly demonstrated the impact of indexing step on the observed relative abundance of taxa at the phylum level starting from one unique sample (Figures 1(a) and 1(b)). On the contrary, Metabiote protocol based on SIM shows a clear greater homogeneity in its results, with no impact of the indexing step (Figure 1(c)). Additionally, Metabiote process gives access to higher bacterial diversity information compared to the two other classical protocols when estimated with the Shannon index [1], as shown for the 48 indexed samples (Figure 2), *p* < 0.01, using the Mann-Whitney statistical analysis. Ten female BALB/c mice (6 weeks old on arrival, Charles River Laboratories, Saint-Germain sur l'Arbresle, France) were housed in a controlled environment (with a temperature of 22°C, a 12 h/12 h light/dark cycle, and* ad libitum* access to food and water) for a minimal acclimatization period of 12 days. All animal experiments were performed according to the guidelines of the* Institut Pasteur de Lille* Animal Care and Use committee and in compliance with the Amsterdam Protocol on Animal Protection and Welfare and Directive 86/609/EEC on the Protections of Animals Used for Experimental and Other Scientific Purposes (updated in the Council of Europe's Appendix A). The animal work was also compliant with French Legislation (the French Act 87-848, dated 19-10-1987) and (the European Communities Amendment of Cruelty to Animals Act 1976). The study's objectives and procedures were approved by the Nord-Pas-de-Calais region's Ethic and Welfare Committee for Experiments on Animals (Lille, France; approval number: 19/2009R). The individual murine fecal samples were freshly collected during defecation, immediately frozen in liquid nitrogen and stored at −80°C until further process. Samples from human feces (1-2 grams in duplicate) from a single healthy volunteer (43 years old, male) were collected at 0, 24, 30, and 48 h time points, quickly frozen, and stored at −80°C. Finally, the single fecal sample of cat origin was taken from the freshly made kitty litter (Globule). All samples were blinded and processed for DNA extraction. Metabiote kit has been used for library preparation according to Genoscreen's recommendations (Genoscreen, Lille, France).

Final libraries each containing 12 different samples identified by a SIM were amplified by emPCR as described in the GS Titanium Amplification Method Manuel Lib-L (http://454.com/downloads/my454/documentation/gs-junior/method-manuals/GSJunior_emPCR_Lib-A_RevApril2011.pdf). Sequencing was performed on a GsFLX Instrument using version 2.9 software. Amplicon libraries were each sequenced on one separate eighth of PicoTiterPlate (PTP) resulting in between 84 000 and 115 000 Passed Filter reads. Read length histogram shows the typical achieved modal read lengths that is in agreement with the Metabiote V3V4 amplicon length. Metabiote OneLine Pipeline has been used to assess microbial population definition, diversity, and comparison. This pipeline comprises the following steps: preprocessing (SIM sorting, no mismatch in specific primer, read length selection, elimination of reads with ambiguous bases, signal quality filter, and homopolymers exclusion), chimera detection, OTU clustering, comparison to the database Greengenes, and taxonomic establishment based on the use of QIIME pipeline [2].

## 3. Results and Comments

**Figure 3:**
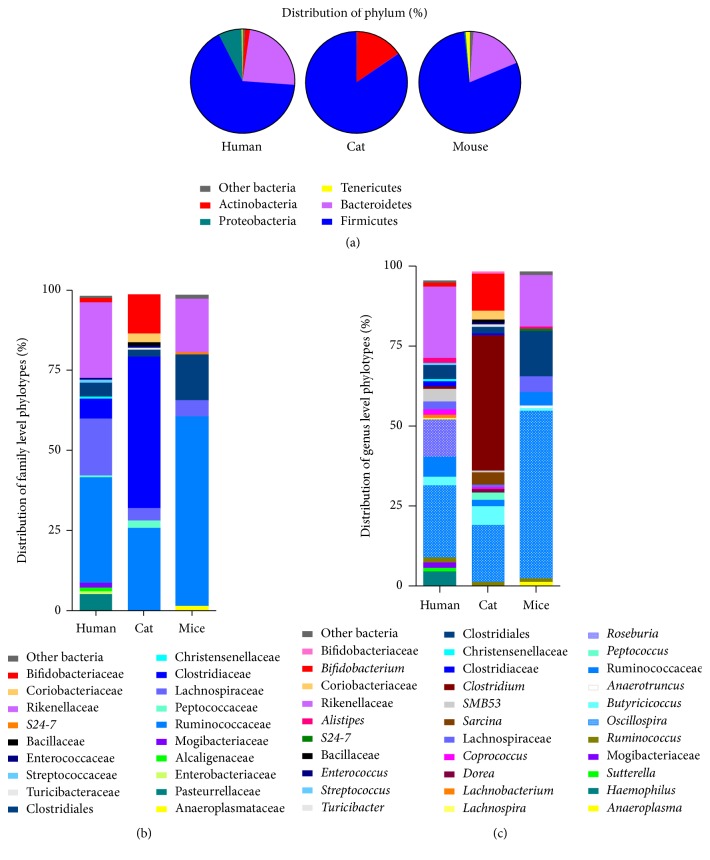
Bacterial community profiling of human, cat, and mouse fecal samples at the phylum (a), family (b), and genus (c) level phylotypes, illustrating possible simultaneous analysis of microbiota assessment of samples from distinct origins.

We first report consistent analysis of samples from distinct origins: human subject, mouse, and cat. A representative example of the corresponding human, cat, and mouse microbial profiles, respectively, obtained at the phylum, family, and genus level is shown in Figure 3. Obviously, the methodology allows identifying highly specific signatures for material from each origin. According to the phylum level, both Firmicutes (over 70%) and Bacteroidetes (20–25%) are detected in mice and man in ranges in agreement with expected results, while Proteobacteria is restricted to a marginal group in mice; the latter is found substantial (10%) in the human subject. Tenericutes were only detected in mice samples. Surprisingly, Gram-negative species are negligibly detected in the cat fecal material where besides the major Firmicutes (85%) Actinobacteria are highly represented (15%). The latter is essentially assigned to* Bifidobacterium* species at the genus level, showing that extremely anaerobic strains are effectively identified. In line, near 50% of the cat bacterial community is made of* Clostridium* species while Clostridiales are part of 5% in human subject and 10% in mice. Data presented here show that methodology allows identifying highly specific signatures for material from each origin. Of note, mice fecal samples appear more similar to human feces than the cat, suggesting a possible use of murine for microbial-related studies and research purposes. However, genetically engineered animals to carry similar microbial profile as man would ideally be desirable. This would require the generation of microbiota-humanized mice with steady and long-term maintenance of the symbiotic communities. So far, no evidence of such complete tolerance has been achieved as some specific human-derived species are probably unable to durably colonize the mouse digestive tract.

**Figure 4:**
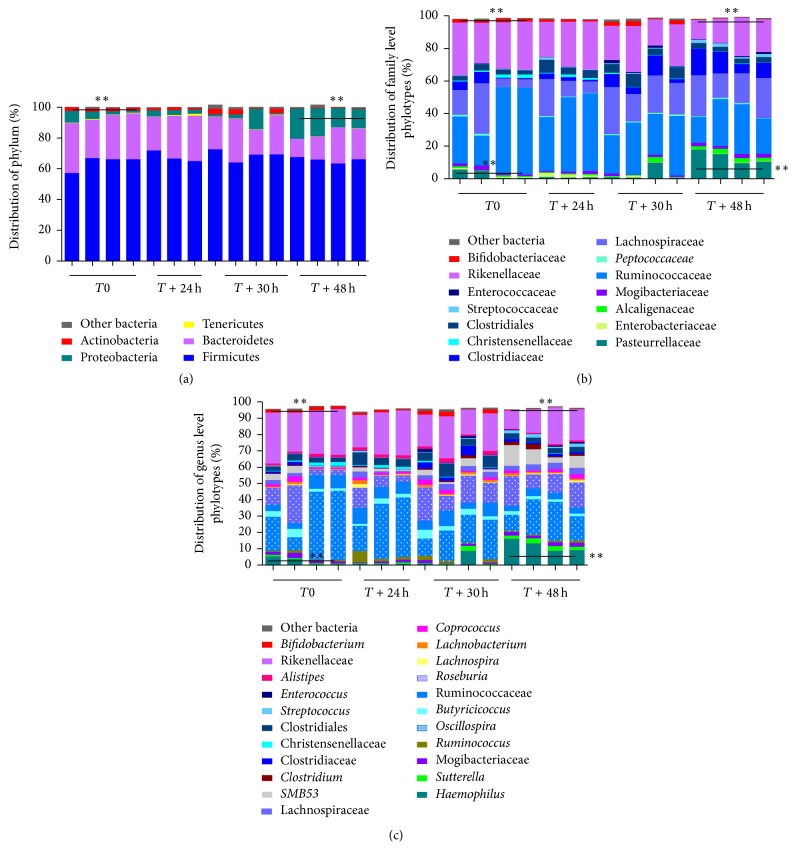
Short term changes in the bacterial community profiling at the phylum (a), family (b), and genus (c) level phylotypes of fecal samples obtained at different time points (0, 24, 30, and 48 h) from a single human subject. For example, a significant increase of Proteobacteria is demonstrated at 48 h compared with *T*0 (^*∗∗*^ *P* < 0.01) whereas Actinobacteria are not detectable any more (^*∗∗*^ *P* < 0.01) (a). These changes are confirmed at lower levels of detection, respectively, Pasteurellaceae (b) and* Haemophilus* (c) and* bifidobateriaceae* (b) and* bifidobacteria* (c) (^*∗∗*^ *P* < 0.01). ^*∗*^Mann-Whitney test, *n* = 4 samples/time points.

We then report human intraindividual variations during a short time course sampling. Structure of the intestinal microbiota varies substantially between individuals [3]. Furthermore, the gut microbiota composition is dynamic and may endure slight variations following the daytime activity, including work habits, sleeping period, and obviously eating varied diets. We collected samples of fecal replicates (two duplicates) from a single human subject at 4 different time points of 0, 24 h, 30 h, and 48 h. As shown in Figure 4, the composition of phylum, family, and genus at the same time point demonstrates minor changes showing that replicates are quite similar. In contrast, more important variations are seen with respect to time. For example, although the core bacterial community is preserved during day time (Rikenellaceae,* Roseburia*, and* Oscillospira*), the microbial profiling is clearly different after 24 h, revealing an increase in* Ruminococcus*. Likewise, analysis at the 48 h time point showed a higher proportion of the phylum Proteobacteria (corresponding to* Haemophilus* spp. from Pasteurellaceae),* Sutterella,* and the clone SMB53 (candidate genus of Clostridiaceae) concomitantly with a drop in Rikenellaceae. Interpreting the sources and consequences of these changes is elusive and mostly speculative here. However, the subtle fluctuations could reasonably be attributed to the direct or indirect impact of ingested food particle and other unidentified activities. Such dynamic has to be considered to conclude to a dysbiotic or stable microbiota and avoid misinterpretation. Indeed, it may allow further stratification of distinct responders both in modeling immune and infectious diseases and for personalized therapeutic interventions.

**Figure 5:**
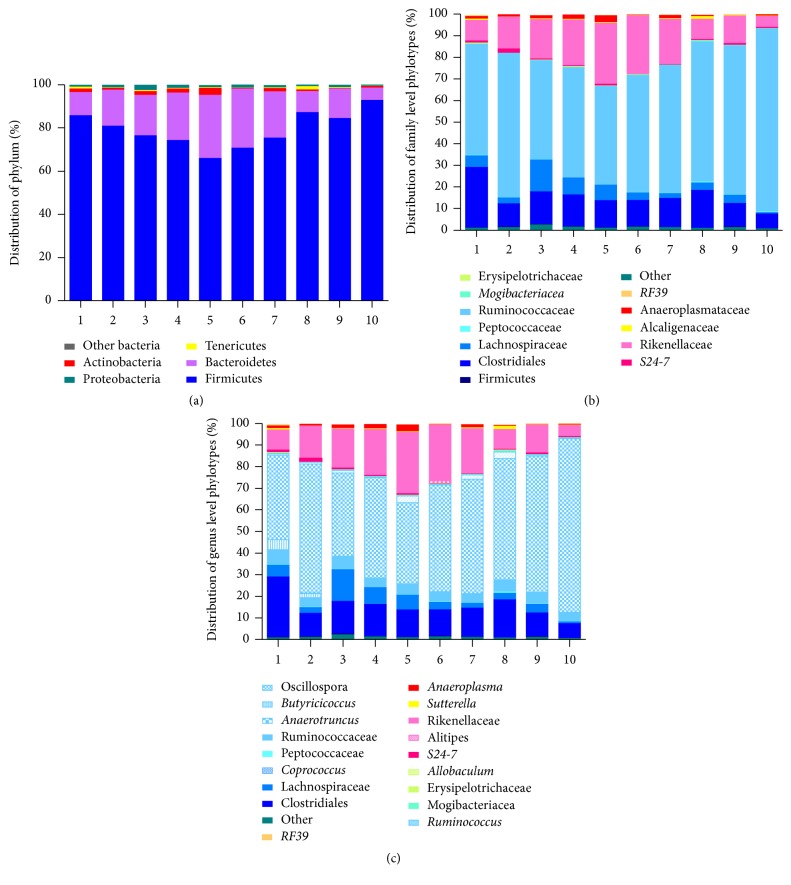
Bacterial community profiling at the phylum (a), family (b), and genus (c) level phylotypes of fecal samples from 10 mice cohoused in the same cage, showing the huge interindividual diversity.

Finally, we addressed the interindividual variations in cohoused mice. A relative uniformity of biological responses is essential in murine experimental models worldwide. Individuality in gut microbiota composition is shaped by complex environmental and host genetic factors [4] and, consequently, variable bacterial communities correspond to specificity in immune and metabolic pathways [5, 6]. The composition (and activities) of intestinal symbiotic microbial consortia highly depends on the mice genetic backgrounds [7] but huge variations between isogenic adult mice reared in different research institutions and providers are observed too [8], as well as important seasonal changes. Moreover, single specificity in mice microbiota profiles may also evoke concerns for research purpose. Here, we questioned the diversity among ten individual mice from the same conventional cage.  Figure 5 illustrates a detailed overview of such individual profiles on the phylum, family, and genus level. Abundance in Firmicutes can represent 60% to 90% while the Bacteroidetes range from 10 to 35%. Less frequent phyla such as Tenericutes and Proteobacteria could or could not be detected. For example,* Ruminococcus* spp. are identified in only 6 mice from the group while four mice are* Alistipes *positive. Such diversity is constantly observed in cagemates from distinct providers upon the arrival and following various diets or treatments (data not shown). Neither coprophagy nor long-term cohousing seems to be able to standardize this fact.

While interindividual variations were previously demonstrated both in mice [9] and in humans, these observations are of great importance in research and should not to be neglected. As far as we can exclude technical bias, intra- and interindividual variations may mask interspecies variations. Our data suggest that minimum 10 mice are required to consider the interindividual variation in the baseline and to exclude possible discrepancies. In addition, it may clearly serve as corner stone for research purposes in microbiota-presumed diseases modeling in rodents, the latter being more realistic and thus fitting the 3Rs ethical rules (replacement, reduction, and refinement) [10]. Although the microbiome science needs a “healthy dose of skepticism” [11], it also requires reliable and consistent tools for gold standard metagenomic analysis.

Collectively, we briefly present a methodology (Metabiote) applied to the microbial profiling of fecal samples from mouse, man, and cat origin. We point out both inter- and intraindividual variations of gut microbial composition in a healthy subject. Knowing the composition of the microbial community alone does not necessarily lead to an understanding of its function. However, such analyses can be helpful to explain time-related changes and discrepancies among animals. Thus, this study suggests the procedure to be useful for diagnostics including dysbiotic states and follow-up of diet and treatments in clinical studies, considering the proper controls are included.
