# Corrigendum to “Snapshot on a Pilot Metagenomic Study for the Appraisal of Gut Microbial Diversity in Mice, Cat, and Man”

**DOI:** 10.1155/2016/3053727

**Published:** 2016-07-28

**Authors:** Coline Plé, Louise-Eva Vandenborght, Nathalie Adele-Dit-Renseville, Jérôme Breton, Catherine Daniel, Stéphanie Ferreira, Benoît Foligné

**Affiliations:** ^1^Université Lille, CNRS, Inserm, CHU Lille, Institut Pasteur de Lille, U1019, UMR 8204, Centre d'Infection et d'Immunité de Lille, 59000 Lille, France; ^2^Genoscreen, Service of Research, Development and Innovation in Health and Environment, 1 rue du Pr Calmette, BP 245, 59019 Lille, France

In the article titled “Snapshot on a Pilot Metagenomic Study for the Appraisal of Gut Microbial Diversity in Mice, Cat, and Man”, [1] the last author's first and last names were reversed. The author's name should have been written Benoît Foligné. The revised author list is shown above.
